# The Aims of Anatomy

**Published:** 1910-01-01

**Authors:** 


### The Hospital

A Journal of

The Medical Sciences and Hospital Administration.

New Series. No. 150, Vol. VI. [No. 1222, Vol. XLVII.] SATURDAY, JANUARY 1, 1910.

### The Aims of Anatomy.

The medical profession has been much exercised lately upon questions of the curriculum and the proper relations of its integral parts. Into the exact proportions which preliminary, biological, physiological, and purely clinical studies should bear to one another in the education of the medical student, we do not propose at the moment to enter, for the matter has frequently been discussed in these columns. Nor, indeed, shall the vexed topic detain us of the exact degree of proficiency in human and animal anatomy which it is necessary and politic for the medical student, the practitioner, or the consulting surgeon respectively to attain. But attention may not unprofitably be directed to a most illuminating introductory lecture delivered at the Royal College of Surgeons, Ireland, by Professor A. Campbell Geddes, the newly-elected successor of the late Professor Fraser. The title of Dr. Geddes' address is that which appears at the head of this page, and the report *in extenso* is to be found in the November issue of the *Dublin Journal of Medical Science*. His contention may be summed up somewhat baldly thus—it is difficult in a narrow compass to do justice to the very far-reaching and stimulating conception he has advanced—that every detail of anatomical structure has some bearing upon function. What that bearing may be, at present is nearly always obscure; but, as the author puts it, "things do not happen just because they happen": an artery or vein, for example, does not appear in an unusual position, save as the result of some pre-existing disturbance of some other part of the developing vascular system. Parenthetically, it may be pointed out that this principle seems at first sight to conflict with one of the two postulates upon which the Darwinian theory is founded, that an ineradicable tendency to variation modifies the influence of heredity. Reflection will show that the inconsistency is more apparent than real.

Anatomy, proceeds Dr. Geddes, is one of the most backward of all the sciences. It has hardly begun to pass beyond the elementary stage of isolated observations. The stage has not yet been reached when, from a study of the surface appearances, we can tell with tolerable certainty the arrangements and proportions of the hidden parts. Yet it is towards this end that anatomy is striving, nor is the struggle, in his belief, hopeless; it is, however, as he sketches it, certainly very arduous and very lengthy. He holds with confidence that there is a definite anatomico-pathological correlation in the structure of the human body which only needs working out to be recognised and acted upon; and he suspects with almost equal confidence that what he calls his "anatomical-pathological system" possesses a clinical extension of vast importance. To make this intelligible he gives us examples which, though little known, are none the less established truths. From the type and method of growth of the hair it is sometimes possible, according to the professor, to estimate accurately the efficiency of the suprarenal bodies and the liver; and from the proportional lengths of the limb bones of an adult it is also possible to judge of the activity of the reproductive glands.

His highly ingenious anatomical theory of tuberculosis, or rather of the tuberculous diathesis, is hardly yet ripe for serious criticism. As a remarkable piece of Celtic and almost poetic deduction it is worth reading, but the staider Saxon will probably prefer his surer, if less brilliant, Baconian methods of scientific investigation. One moral may at least be emphasised, and for drawing attention to it Professor Geddes may be most cordially thanked. It is the impolitic and immoral divorce of anatomy from physiology, pathology, and clinical medicine. Anatomists, he admits, are too prone to dissociate themselves from their medical brethren, and too much inclined to think of anatomy as all-embracing: too little anxious to welcome the clinician and the pathologist in the dissecting room. He looks forward to a New Preventive Medicine, based in part upon a New Anatomy. A Medicine that, acting on the tactical principle known to military men as the Offensive-Defensive, goes out to meet its foe, Disease. Thus, among other anatomico-pathological observations, he has noticed an unusual development of subcutaneous veins across the sternum in the subjects of pulmonary tuberculosis. Believing that this is not a consequence of the disease, but rather a cause, or at any rate the result of some other abnormality which renders vulnerable the individual possessing it, he would lay down a rule that any such patient, who can be shown to possess also any other anatomical peculiarity which is evidence of predisposition to morbidity, should be treated in some way to prevent tuberculosis. This is Professor Geddes' conception of the aim of scientific anatomy. It is a lofty ideal, for it involves the advance from a purely descriptive into an inductive science. The anatomist of the future must break with the somewhat pedagogic past which has repelled so many students in the dissecting room. He must consciously regard the tissues as adaptable changing things, which can be moulded by the intelligence of the supervising physician. It is an ambition which may not, almost certainly will not, be realised in Dr. Geddes' day or in that of his youngest student; but it is one which must have a stimulating effect on those who work under this farseeing and greatly daring innovator. We trust its author may live to see great progress made in the direction he has pointed out.

